# News from *Arabidopsis* on the Meiotic Roles of Blap75/Rmi1 and Top3α

**DOI:** 10.1371/journal.pgen.1000306

**Published:** 2008-12-19

**Authors:** Charles I. White

**Affiliations:** Génétique, Reproduction, et Développement, CNRS UMR 6247, Clermont Université, INSERM U931, Aubière Cedex, France

Two articles published in this issue of *PLoS Genetics* present novel data concerning the members of a key regulator of genetic crossing-over. Working with the plant *Arabidopsis thaliana*, the authors of the two reports provide exciting new data and further understanding of the meiotic anti–crossing-over function of the Topoisomerase 3alpha (Top3α) and Blap75/Rmi1 proteins, and thus presumably that of the protein complex that contains these proteins and the RecQ-like helicase BLM.

The highly conserved RecQ-like helicase BLM, which is mutated in patients with Bloom syndrome, acts in a protein complex that can disassociate homologous recombination intermediates in vitro and in vivo (reviewed in [1]–[3]). The importance of this anti-recombination role is clearly shown by the elevated levels of genetic instability, mitotic recombination, and sister-chromatid exchanges in the somatic tissues of the cancer-prone Bloom syndrome patients. This complex, known as BTB in mammals and RTR in yeast, involves BLM and at least two other proteins: Top3α and Blap75/Rmi1. BLAP75/RMI1 is a highly conserved protein in eukaryotes originally identified through its interactions with the BLM [4],[5] and independently as Rmi1/Nce4 in yeast through its genetic interactions with BLM homolog Sgs1 [1],[6]. A fourth protein component of this complex, Rmi2, has recently been identified [7],[8], and it is likely that others will follow (discussed by [9]). It is proposed that the principal anti-recombinational role of this complex involves BLM helicase-driven migration of double Holliday junctions (dHJs) to form a hemi-catenane intermediate. The resolution of this structure by the action of a topoisomerase (Top3α) does not lead to the exchange of flanking DNA sequences, and thus BLM acts to avoid crossing-over [3], [10]–[12]. BLM also has affinity for DNA structures other than dHJ and clearly also plays other anti-recombination roles [13]–[15]. To add to these, a very recent report shows a pro-recombination role for Sgs1/BLM in resection of 5′-ended strands at DNA double-strand breaks [16].

What about *A. thaliana*, the subject of the two reports discussed here? *Arabidopsis* has a total of seven identified RecQ-like proteins, with RecQ4a being the strongest candidate for the *Arabidopsis* BLM/Sgs1 ortholog [17]–[19]. The accompanying papers report the identification of the *Arabidopsis* othologs of BLAP75/Rmi1 [20],[21] and Topo3α [21], as well as the characterization of the mitotic and meiotic phenotypes of the corresponding mutant plants. *top3*α mutant plants have severe developmental defects, are methyl methanesulfonate (MMS)–sensitive, and show elevated levels of mitotic recombination and mitotic chromosome abnormalities. Similar mitotic phenotypes are observed in *recQ4a* and *blap75/rmi1* mutant plants, suggesting a functional interaction between RecQ4a and Top3α. This is further supported by the partial suppression of *top3*α developmental defects in double *recQ4a/top3*α mutants.

In most (studied) eukaryotes, homologous recombination that occurs during the first meiotic prophase ensures the proper segregation of homologous chromosomes (homologs) at the first meiotic division. These events are initiated by programmed double-strand breaks that generate broken DNA ends that invade homologous sequences on the homolog, a subset of which are processed to form dHJs. These must be resolved to permit the separation of homologs at the first meiotic anaphase, and the mode of this resolution determines whether or not the recombination is accompanied by physical exchange of chromosome arms of the homologs (crossing-over). The potential of crossing-over to cause genome reorganization (insertions, deletions, inversions, translocations) has led to the evolution of multiple controls of recombination.

It has long been recognized that the numbers and distribution of meiotic cross-overs are strictly regulated. In the last decade, the existence of cross-over and non–cross-over recombination pathways has been established, and many details of molecular mechanisms elucidated [22]–[28]. In this context lies the importance of the characterization of the essential meiotic anti–crossing-over role of the BTB/RTR complex in *Arabidopsis* by the Grelon and Puchta groups, reported in this issue of *PLoS Genetics* [20],[21].

These reports show that *Arabidopsis blap75/rmi1* and *top3*α mutants are capable of full chromosome synapsis, resulting in normal pachytene figures. The structure of the synaptonemal complex at pachytene was verified by staining of *blap75* mutant meioses with antisera against Asy1 and Zyp1, two synaptonemal complex proteins, and proper chromosome pairing was shown by fluorescence in situ hybridization (FISH) [20]. Staining with antiserum against Dmc1, a marker for early meiotic recombination intermediates, also shows normal numbers and timing of foci. Although these immunological and FISH analyses haven't been carried out for *top3*α mutants, the DAPI-stained pachytene figures of *top3*α present the same (normal) aspect as those of the *blap75* mutants. Epistasis analyses confirm that Blap75/Rmi1 acts downstream of Spo11 (DNA cleavage/recombination initiation), Rad51, and Mnd1 (homolog invasion). It thus appears that early steps of meiosis, up to homolog pairing and synaptonemal complex formation, occur normally in the absence of Blap75/Rmi1 and Top3α in *Arabidopsis*. However, aberrant diakinesis and interlocked metaphase I figures follow, and chromosomes fragment at snaphase I. The interlocked bivalents observed at diakinesis and metaphase I may be due to the presence of the unresolved recombination intermediates, including those between more than two chromatids that are seen in yeast [29],[30]. The fragmentation of univalent chromosomes at meiotic anaphase I in double *dmc1/blap75* mutants might also suggest such interchomatid linkages (*Arabidopsis dmc1* mutants have asynaptic meiosis [31]), although other explanations, such as the existence of unrepaired chromatid breaks, are equally likely. Interestingly, no later meiotic stages (second division) were seen in either study, implying meiotic arrest at the end of the first division. This phenotype contrasts strikingly with that seen in many *Arabidopsis* recombination mutants, such as *rad50* or *rad51*, which complete the two meiotic divisions, notwithstanding severe chromosome fragmentation at leptotene [32],[33]. The dependence of this meiosis I arrest upon the presence of paired chromosomes is confirmed by the completion of the two meiotic divisions in double *dmc1/blap75* mutants. Homologous chromosome recognition and synapsis through recombination thus progresses to bivalent formation in *blap75/rmi1* mutants, even in the absence of the ZMM proteins Mer3 and Msh5—notwithstanding the fact that they are required for the major meiotic cross-over pathway in *Arabidopsis* [34]–[36]. The bivalents of *blap75/rmi1* mutants are, however, interlinked and unable to separate properly, finally fragmenting at anaphase I.

Blap75/Rmi1 and Top3α are thus needed for resolution of recombination intermediates that form between both sister chromatids and homologs, and are essential for separation of bivalents and meiosis I chromosomal disjunction in *Arabidopsis*. An essential meiosis I role is also seen in yeast *top3* and *rmi1* mutants [1],[37]. However, *Arabidopsis recQ4a* mutants have apparently normal meiosis and are fertile, as are mouse *BLM* ^−/−^ mutants, and yeast *sgs1* mutants show only minor defects in meiosis [38],[40]. Thus, Blap75/Rmi1 and Top3α appear to have meiotic functions that are independent of RecQ4a. The relative high fertility of yeast *sgs1* mutants may be explained by findings that Sgs1 and the Mus81/Mms4 nuclease can partially substitute for each other [29],[30]. The co-lethality of *recQ4a* and *mus81* in *Arabidopsis* [39] is suggestive of a similar situation in mitosis in yeast, although their relation in meiosis remains to be determined.

These findings lead to the question of whether the critical meiotic function of Blap75/Rmi1 and Top3α in resolving recombination intermediates is performed by these two proteins alone, or whether they perform this function in complex with another helicase. Other unanswered questions concern the nature of the meiotic joint molecule intermediates, the resolution of which requires Blap75/Rmi1 and Top3α in *Arabidopsis*. Does the absence of these proteins lead to an excess of normal dHJs, overwhelming the capacity of other HJ resolvase(s), or are these aberrant, unresolvable structures? The Mus81 nuclease acts in an interference-insensitive cross-over pathway in *Arabidopsis* meiosis [41], but clearly it cannot complement the absence of Blap75/Rmi1 or Top3α—what role does it play in recombination intermediate metabolism in *Arabidopsis* meiosis?

In addition to their importance for the understanding of recombination and meiosis in plants, these results extend the known meiotic activities of this complex—demonstrating and clearly placing its essential role in the separation of synapsed chromosomes at the first meiotic division.

## References

[1] Chang M, Bellaoui M, Zhang C, Desai R, Morozov P (2005). RMI1/NCE4, a suppressor of genome instability, encodes a member of the RecQ helicase/Topo III complex.. EMBO J.

[2] Lohman TM, Tomko EJ, Wu CG (2008). Non-hexameric DNA helicases and translocases: mechanisms and regulation.. Nat Rev Mol Cell Biol.

[3] Wu L, Hickson ID (2003). The Bloom's syndrome helicase suppresses crossing over during homologous recombination.. Nature.

[4] Meetei AR, Sechi S, Wallisch M, Yang D, Young MK (2003). A multiprotein nuclear complex connects Fanconi anemia and Bloom syndrome.. Mol Cell Biol.

[5] Yin J, Sobeck A, Xu C, Meetei AR, Hoatlin M (2005). BLAP75, an essential component of Bloom's syndrome protein complexes that maintain genome integrity.. EMBO J.

[6] Mullen JR, Nallaseth FS, Lan YQ, Slagle CE, Brill SJ (2005). Yeast Rmi1/Nce4 controls genome stability as a subunit of the Sgs1-Top3 complex.. Mol Cell Biol.

[7] Singh TR, Ali AM, Busygina V, Raynard S, Fan Q (2008). BLAP18/RMI2, a novel OB-fold-containing protein, is an essential component of the Bloom helicase-double Holliday junction dissolvasome.. Genes Dev.

[8] Xu D, Guo R, Sobeck A, Bachrati CZ, Yang J (2008). RMI, a new OB-fold complex essential for Bloom syndrome protein to maintain genome stability.. Genes Dev.

[9] Liu Y, West SC (2008). More complexity to the Bloom's syndrome complex.. Genes Dev.

[10] Bussen W, Raynard S, Busygina V, Singh AK, Sung P (2007). Holliday junction processing activity of the BLM-Topo IIIalpha-BLAP75 complex.. J Biol Chem.

[11] Raynard S, Bussen W, Sung P (2006). A double Holliday junction dissolvasome comprising BLM, topoisomerase IIIalpha, and BLAP75.. J Biol Chem.

[12] Wu L, Bachrati CZ, Ou J, Xu C, Yin J (2006). BLAP75/RMI1 promotes the BLM-dependent dissolution of homologous recombination intermediates.. Proc Natl Acad Sci U S A.

[13] Bachrati CZ, Borts RH, Hickson ID (2006). Mobile D-loops are a preferred substrate for the Bloom's syndrome helicase.. Nucleic Acids Res.

[14] Bugreev DV, Yu X, Egelman EH, Mazin AV (2007). Novel pro- and anti-recombination activities of the Bloom's syndrome helicase.. Genes Dev.

[15] Mankouri HW, Ngo HP, Hickson ID (2007). Shu proteins promote the formation of homologous recombination intermediates that are processed by Sgs1-Rmi1-Top3.. Mol Biol Cell.

[16] Gravel S, Chapman JR, Magill C, Jackson SP (2008). DNA helicases Sgs1 and BLM promote DNA double-strand break resection.. Genes Dev.

[17] Bagherieh-Najjar MB, de Vries OM, Hille J, Dijkwel PP (2005). Arabidopsis RecQI4A suppresses homologous recombination and modulates DNA damage responses.. Plant J.

[18] Hartung F, Plchová H, Puchta H (2000). Molecular characterisation of RecQ homologues in Arabidopsis thaliana.. Nucleic Acids Res.

[19] Hartung F, Suer S, Puchta H (2007). Two closely related RecQ helicases have antagonistic roles in homologous recombination and DNA repair in Arabidopsis thaliana.. Proc Natl Acad Sci U S A.

[20] Chelysheva L, Vezon D, Belcram K, Gendrot G, Grelon M (2008). The Arabidopsis BLAP75/RMI1 homologue plys crucial roles in meiotic double strand break repair.. PLoS Genet.

[21] Hartung F, Suer S, Knoll A, Wurz-Wildersinn R, Puchta H (2008). Topoisomerase 3a and RMI1 Are Not Only Required for the Suppression of Somatic Crossovers but Are Also Essential for the Resolution of Meiotic Recombination Intermediates in Arabidopsis thaliana.. PLoS Genet.

[22] Allers T, Lichten M (2001). Differential timing and control of noncrossover and crossover recombination during meiosis.. Cell.

[23] Berchowitz LE, Copenhaver GP (2008). Division of labor among meiotic genes.. Nat Genet.

[24] Bishop DK, Zickler D (2004). Early decision; meiotic crossover interference prior to stable strand exchange and synapsis.. Cell.

[25] Borner GV, Kleckner N, Hunter N (2004). Crossover/noncrossover differentiation, synaptonemal complex formation, and regulatory surveillance at the leptotene/zygotene transition of meiosis.. Cell.

[26] Hunter N, Kleckner N (2001). The single-end invasion: an asymmetric intermediate at the double-strand break to double-holliday junction transition of meiotic recombination.. Cell.

[27] Jones GH, Franklin FC (2006). Meiotic crossing-over: obligation and interference.. Cell.

[28] Mezard C, Vignard J, Drouaud J, Mercier R (2007). The road to crossovers: plants have their say.. Trends Genet.

[29] Jessop L, Lichten M (2008). Mus81/Mms4 endonuclease and Sgs1 helicase collaborate to ensure proper recombination intermediate metabolism during meiosis.. Mol Cell.

[30] Oh SD, Lao JP, Taylor AF, Smith GR, Hunter N (2008). RecQ helicase, Sgs1, and XPF family endonuclease, Mus81-Mms4, resolve aberrant joint molecules during meiotic recombination.. Mol Cell.

[31] Couteau F, Belzile F, Horlow C, Grandjean O, Vezon D (1999). Random chromosome segregation without meiotic arrest in both male and female meiocytes of a dmc1 mutant of Arabidopsis.. Plant Cell.

[32] Bleuyard J, Gallego ME, White CI (2004). Meiotic defects in the Arabidopsis rad50 mutant point to conservation of the MRX complex function in early stages of meiotic recombination.. Chromosoma.

[33] Li W, Chen C, Markmann-Mulisch U, Timofejeva L, Schmelzer E (2004). The Arabidopsis AtRAD51 gene is dispensable for vegetative development but required for meiosis.. Proc Natl Acad Sci U S A.

[34] Higgins J, Vignard J, Mercier R, Pugh AG, Franklin FC (2008). AtMSH5 partners AtMSH4 in the class I meiotic crossover pathway in Arabidopsis thaliana, but is not required for synapsis.. Plant J.

[35] Lu X, Liu X, An L, Zhang W, Sun J (2008). The Arabidopsis MutS homolog AtMSH5 is required for normal meiosis.. Cell Res.

[36] Mercier R, Jolivet S, Vezon D, Huppe E, Chelysheva L (2005). Two meiotic crossover classes cohabit in Arabidopsis: one is dependent on MER3,whereas the other one is not.. Curr Biol.

[37] Gangloff S, de Massy B, Arthur L, Rothstein R, Fabre F (1999). The essential role of yeast topoisomerase III in meiosis depends on recombination.. EMBO J.

[38] Watt PM, Louis EJ, Borts RH, Hickson ID (1995). Sgs1: a eukaryotic homologue of E. coli RecQ that interacts with topoisomerase II in vivo and is required for faithful chromosome segregation.. Cell.

[39] Hartung F, Suer S, Bergmann T, Puchta H (2006). The role of AtMUS81 in DNA repair and its genetic interaction with the helicase AtRecQ4A.. Nucleic Acids Res.

[40] Luo G, Santoro IM, McDaniel LD, Nishijima I, Mills M (2000). Cancer predisposition caused by elevated mitotic recombination in Bloom mice.. Nat Genet.

[41] Berchowitz LE, Francis KE, Bey AL, Copenhaver GP (2007). The role of AtMUS81 in interference-insensitive crossovers in A. thaliana.. PLoS Genet.

